# The value of a combined model based on ultra-radiomics and multi-modal ultrasound in the benign-malignant differentiation of C-TIRADS 4A thyroid nodules: a prospective multicenter study

**DOI:** 10.3389/fonc.2025.1543020

**Published:** 2025-05-08

**Authors:** Shuai Cui, Qifan Liu, Hailong Wang, Husha Li, Wei Li, Chenlong Li, Leilei Bi, Yang Mu, Wenjing Guo, Jundong Yao, Zhoulong Zhang

**Affiliations:** ^1^ Department of Ultrasound, The First Affiliated Hospital, College of Clinical Medicine of Henan University of Science and Technology, Luoyang, China; ^2^ Department of Ultrasound, General Hospital of Pingmei Shenma Medical Group, Pingdingshan, China; ^3^ Department of Ultrasound, Xiangyang Hospital of Traditional Chinese Medicine, Xiangyang, China; ^4^ Department of Ultrasound, The Fourth Affiliated Hospital of Xinjiang Medical University, Urumqi, China

**Keywords:** C-TIRADS 4A, multimodal ultrasound, ultrasound radiomics, papillary thyroid cancer, benign or malignant

## Abstract

**Objective:**

To establish a combined model based on ultrasound radiomics combined with multimodal ultrasound and evaluate its value in diagnosing benign and malignant nodules classified as Chinese-Thyroid Imaging Report and Data System (C-TIRADS) 4A.

**Methods:**

Prospective collection of data from 446 patients with thyroid nodules classified as C-TIRADS 4A between December 2023 and August 2024. Based on the enrollment timeline, patients were divided into a training set (n=312) and a test set (n=134) in a 7:3 ratio. Using clinical information, multimodal ultrasound features, and radiomics features, a radiomics model was constructed using the Random Forest (RF) machine learning algorithm. Logistic regression was employed to develop the multimodal ultrasound model and the combined model. The predictive efficiency and accuracy of these models were evaluated using Receiver Operating Characteristic (ROC) curves, calibration curves, and Decision Curve Analysis (DCA). The diagnostic efficacy of junior physicians assisted by the ultrasound radiomics model was compared with that of senior physicians. DeLong’s test was performed to compare the diagnostic performance of the models.

**Results:**

Multivariate analysis revealed that age (≤51 years), Sound Touch Elastography mean stiffness (STE Mean), orientation (vertical), margin (blurred), and margin (irregular) were independent risk factors for papillary thyroid carcinoma, and the multimodal ultrasound model was established. Based on 17 ultrasound radiomics features, a radiomics model was constructed using the RF machine learning algorithm. The combined model was developed by combining the two aforementioned models. In the training set, the areas under the curve (AUC) of the multimodal ultrasound model, ultrasound radiomics model, and combined model were 0.852, 0.940 and 0.956, respectively. In the test set, the AUC were 0.804, 0.832 and 0.863, respectively. DeLong’s test showed that the combined model performed best in the training set, and in the test set, the combined model outperformed the multimodal ultrasound model but showed no significant difference compared to the radiomics model. DCA indicated that the combined model achieved higher net benefits within a specific threshold probability range (0.15-0.90).

**Conclusion:**

The combined model exhibits robust diagnostic capability in distinguishing benign from malignant thyroid nodules classified as C-TIRADS 4A.

## 1 Introduction

Thyroid cancer is one of the fastest-growing malignancies in terms of incidence worldwide. Over the past three decades, its global incidence has risen significantly, currently ranking as the seventh most common cancer globally (1, 2). Papillary thyroid cancer (PTC) accounts for more than 80% of all thyroid cancer cases (3). Although its prognosis is relatively favorable, the issue of overtreatment during diagnosis and management has become increasingly prominent.

Ultrasound examination has become the preferred imaging tool for risk assessment of thyroid nodules due to its non-invasive, real-time, and reproducible advantages. The establishment of the Chinese-Thyroid Imaging Report and Data System (C-TIRADS) has provided an important framework for standardizing the risk assessment of thyroid nodule malignancy (4). However, the malignancy risk of C-TIRADS 4A nodules spans a relatively wide range (2%-10%) (5, 6), leading to a significant number of patients undergoing unnecessary fine-needle aspiration biopsies or surgeries due to diagnostic uncertainty. This not only increases healthcare costs but may also cause patient anxiety. Therefore, there is an urgent need for a more precise differentiation method to optimize the management strategy for C-TIRADS 4A nodules.

Currently, the traditional C-TIRADS primarily relies on the morphological features of grayscale ultrasound (such as margins, echogenicity, calcifications, etc.) for risk stratification. However, the introduction of multimodal ultrasound techniques, including elastography, Color Doppler Flow Imaging (CDFI), and contrast-enhanced ultrasound, has provided multidimensional information for assessing nodule stiffness, vascular characteristics, and microcirculation. For example, Gong et al. (7) demonstrated that combining grayscale ultrasound with contrast-enhanced ultrasound improved the diagnostic the areas under the curve (AUC) from 0.844 to 0.897. Additionally, Nattabi et al. (8) further showed that nodule stiffness measured by Shear Wave Elastography (SWE) is significantly positively correlated with malignancy risk. Nevertheless, these techniques still heavily depend on physician experience, and there are notable differences in diagnostic consistency among physicians of varying experience levels, with the learning curve for junior physicians being particularly challenging.

The emergence of radiomics offers a novel approach to addressing the aforementioned challenges. This technology extracts high-throughput deep imaging features (such as texture, morphology, heterogeneity, etc.) and combines them with machine learning algorithms to construct objective quantitative models. It has already demonstrated remarkable potential in the differential diagnosis of tumors such as breast cancer and liver cancer (9–11). In the field of thyroid imaging, the application of ultrasound radiomics is still in its exploratory stages. However, preliminary studies suggest that it can effectively capture malignant features that are difficult to identify using traditional methods, such as microstructural heterogeneity within nodule (12). Nevertheless, the complementary nature of multimodal ultrasound parameters (e.g., vascular characteristics, elasticity modulus) and radiomics features has not yet been fully explored, and there is a lack of validation based on multicenter data.

This study aims to develop a machine learning model that integrates multimodal ultrasound features and ultrasound radiomics for the differentiation of benign and malignant thyroid nodules classified as C-TIRADS 4A. Through prospective multicenter data validation, the diagnostic performance of the model and its potential to assist junior physicians will be evaluated. The goal is to reduce unnecessary invasive procedures, shorten the learning curve for junior physicians, and enhance their diagnostic capabilities, thereby optimizing clinical decision-making processes.

## 2 Materials and methods

### 2.1 Study population

**Figure 1:**
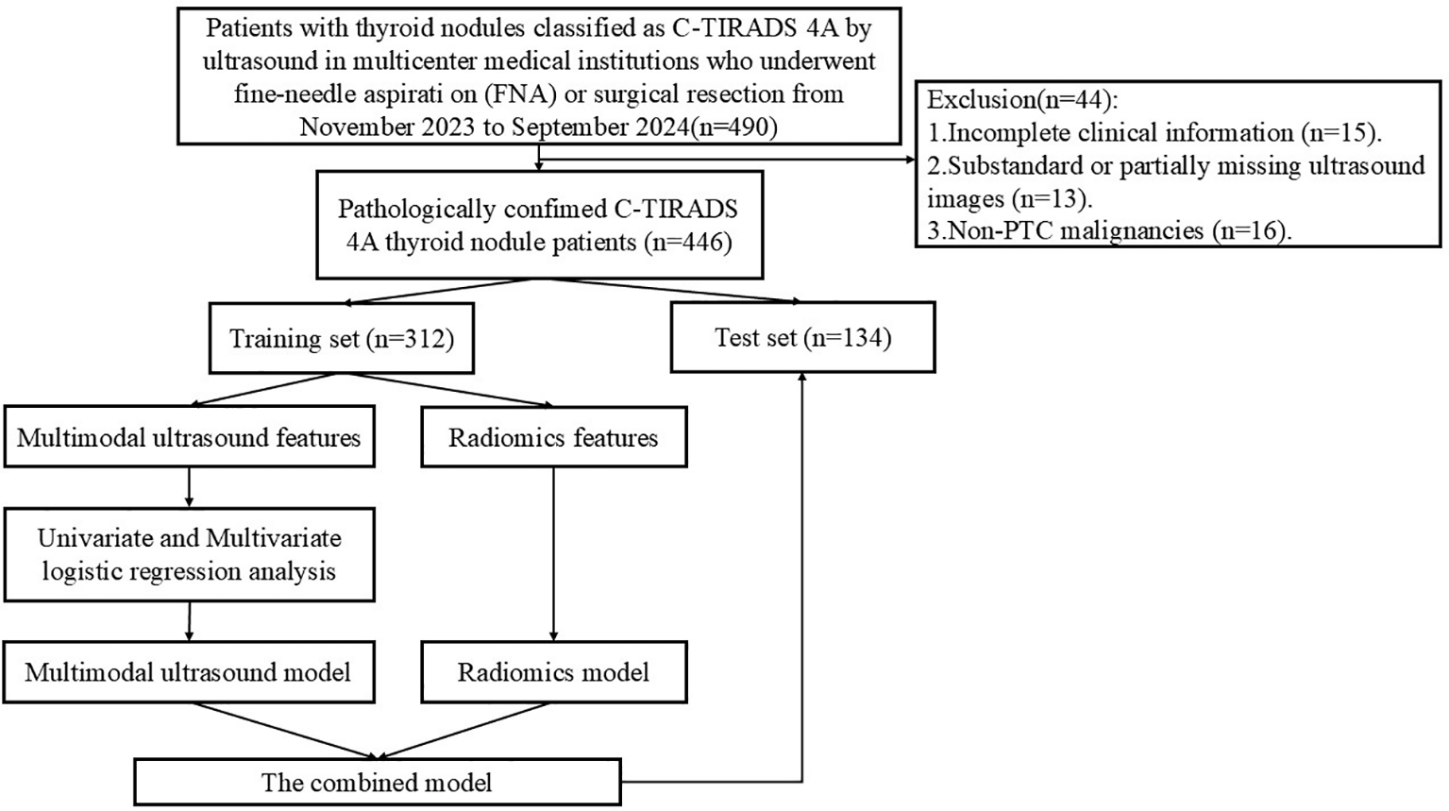
Flowchart of patient enrollment.

Prospectively collect data from patients with thyroid nodules classified as C-TIRADS 4A by ultrasound in multicenter medical institutions from December 2023 to August 2024. The inclusion criteria were as follows: ① age ≥ 18 years; ② ultrasound diagnosis of single or multiple C-TIRADS 4A nodules; ③ undergoing fine-needle aspiration biopsy (FNA) combined with BRAF V600E gene testing or surgical resection with definitive pathological results. The exclusion criteria were as follows: ① poor-quality ultrasound images (suboptimal image quality or insufficient resolution); ② history of neck surgery, cancer, or pregnancy; ③ incomplete clinical information; ④ non-PTC malignancies. A total of 446 patients were ultimately included, with 314 cases from The First Affiliated Hospital of Henan University of Science and Technology, 53 cases from General Hospital of Pingmei Shenma medical group, 48 cases from Xiangyang Hospital of Traditional Chinese Medicine, and 31 cases from The Fourth Affiliated Hospital of Xinjiang Medical University. Based on the enrollment timeline, patients were divided into a training set and a test set in a 7:3 ratio. **Figure 1** illustrates the patient enrollment flowchart. This study received approval from the Ethics Committee of the First Affiliated Hospital of Henan University of Science and Technology (2024-03-K0160), All participants provided written informed consent, in compliance with regulations of the institution and the guidelines of the Declaration of Helsinki.

### 2.2 Ultrasound image acquisition

Prior to data collection, all ultrasound physician underwent centralized training on C-TIRADS 4A classification criteria, including grayscale ultrasound features (e.g., margins, echogenicity, calcifications) and multimodal parameters (e.g., elastography thresholds). All participating centers utilized identical ultrasound systems (Mindray Resona I9) equipped with linear array probes (frequency range: 7.5–12 MHz). Imaging parameters were standardized across centers, including gain (60–70 dB), depth (2.5-3.5 cm), dynamic range (50–60 dB), and mechanical index (MI: 0.8-1.0). The patient was positioned supine, with the neck fully extended to expose the thyroid area. Grayscale images, color Doppler images, and dynamic images of nodules were collected in both transverse and longitudinal sections. Elastography settings were uniformly configured to ensure consistent Young’s modulus calculations. Subsequently, three types of elastography images were acquired: Sound Touch Elastography (STE), Strain Elastography (SE), and Sound Touch Quantification (STQ). All images are stored in DICOM format.

### 2.3 Ultrasound image analysis

Ultrasound image feature was conducted by two doctors, Jundong Yao and Wei Li, both possessing 10 years of experience in ultrasonic diagnosis of thyroid diseases. They independently analyzed the images without access to the patients’ clinical information or pathological results. In cases of differing opinions, a third doctor, Zhoulong Zhang, who has over 30 years of experience in ultrasound diagnosis, made the final decision. This study evaluates several features of nodules, including maximum diameter of the nodule, multiplicity (solitary/multiple), margin (smooth/ill-defined/irregular), halo sign (present/absent), composition (cystic-solid/solid/predominantly solid), echogenicity (isoechoic/markedly hypoechoic/hypoechoic/hyperechoic/heterogeneous), echo texture (homogeneous/heterogeneous), orientation (vertical/parallel), calcification (absent/coarse calcification/microcalcification/indeterminate punctate echogenic foci/peripheral calcification), relationship to capsule (>2 mm/≤2 mm/extracapsular extension), and CDFI classification according to Alder (levels 0/I/II/III). The region of interest (ROI) for elastography is defined by manually tracing the contours of nodules using a specialized machine. The shell denotes a machine-generated boundary that extends 2 mm beyond the nodule following the tracing process. By outlining the nodule area, the system automatically calculates the STE mean stiffness (STE Mean), STE maximum stiffness (STE Max), STE minimum stiffness (STE Min), STE standard deviation (STE SD), SE mean stiffness (SE Mean), SE maximum stiffness (SE Max), SE minimum stiffness (SE Min), SE standard deviation (SE SD), STQ mean stiffness (STQ Mean), STQ maximum stiffness (STQ Max), STQ minimum stiffness (STQ Min), STQ standard deviation (STQ SD).

### 2.4 Pathological analysis

Ultrasound-guided fine-needle aspiration biopsy was performed by ultrasonologists who had completed standardized training and obtained qualification certificates. A 23G fine needle was utilized to efficiently extract samples from the thyroid lesion under ultrasound guidance. The cells were preserved in a liquid-based culture medium for subsequent genetic analysis. The test samples were then sent for pathological examination and genetic testing. A pathological diagnosis of PTC is deemed positive. The BRAF V600E mutation (a key genetic driver of PTC) is strongly associated with tumor aggressiveness, extrathyroidal extension, and lymph node metastasis (13). Its detection enhances diagnostic specificity for malignancy. If the BRAF V600E mutation is detected, it is recorded as positive; if not, it is noted as negative. All results were followed up for 6 months.

### 2.5 Model building

#### 2.5.1 Construction of the multimodal ultrasound model

Univariate logistic regression analysis was conducted on the selected variables, which included gender, age, and ultrasound image characteristics such as STE Mean, STE Max, STE Min, STE SD, SE Mean, SE Max, SE Min, SE SD, STQ Mean, STQ Max, STQ Min, STQ SD, maximum diameter of the nodule, multiplicity, margin, halo sign, composition, echogenicity, echo texture, orientation, calcification, relationship to capsule and CDFI of the nodule. Variables with a *p-value* < 0.05 were considered risk factors. Subsequently, a multi-factor logistic regression analysis was performed, and independent risk factors were identified through stepwise forward logistic regression to construct a multimodal ultrasound model (Multi-model).

#### 2.5.2 Construction of the ultrasound radiomics model

**Figure 2:**
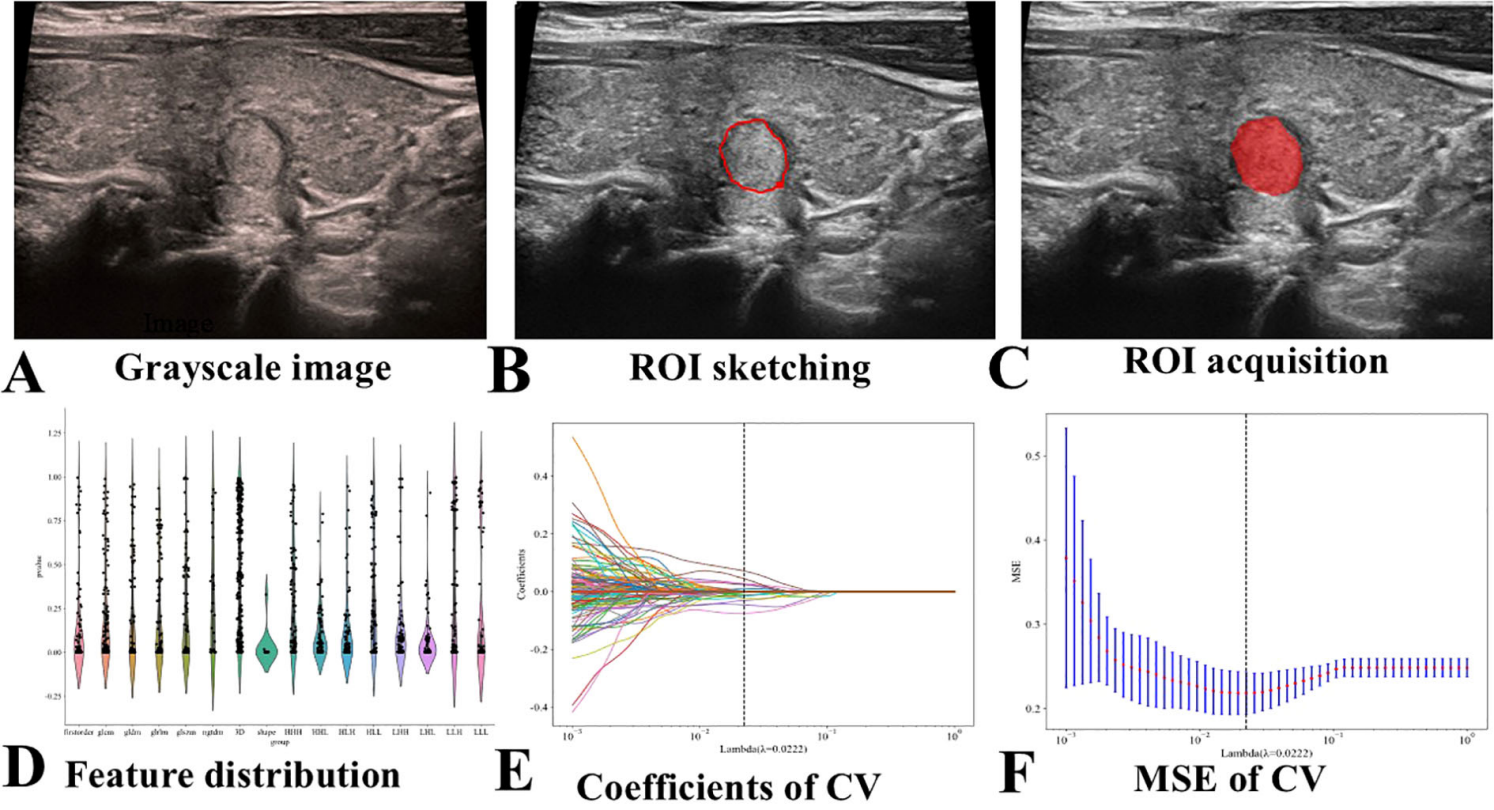
Flowchart of Ultrasound Radiomics Model Construction. CV stands for cross-validation, and MSE stands for mean square error. **(A)** Selection of representative thyroid nodule images. **(B)** Manual delineation of the ROI. **(C)** Example of a segmented ROI after manual delineation. **(D)** Normalization of the extracted radiomics features to conform to a standard normal distribution. **(E)** Application of LASSO regression for feature selection. **(F)** Final selection of radiomics features after dimensionality reduction.

An ultrasound physician with 5 years of diagnostic experience (Husha Li) selected representative thyroid nodule images from each patient who met the inclusion criteria, utilizing the RadiAnt DICOM Viewer 2021.1 software (Medixant, Poznan, Poland), and saved the selected images in DICOM format (**Figure 2A**). Another ultrasound physician with 5 years of experience in ultrasound diagnosis, Hailong Wang, manually depicted the ROI images of the selected patients using the polygon mode in ITK-SNAP software (www.itksnap.org) without understanding the pathological results (**Figures 2B, C**). The DICOM images and segmented ROIs were subsequently imported into the radiomics software PyRadiomics for feature extraction, resulting in radiomics feature. The extracted radiomics features were normalized to conform to a N ~ (0, 1) distribution (**Figure 2D**). The Spearman correlation coefficient was employed to assess the correlation between features. For features exhibiting a correlation coefficient greater than 0.9, only one of the correlated features was retained, yielding radiomics feature selection. LASSO regression was applied for cross-validation and to determine the optimal penalty coefficient lambda. Features with a zero coefficient were excluded, and further dimensionality reduction was conducted to derive final radiomics features (**Figures 2E, F**). The features with non-zero coefficients were aggregated into a formula to compute the final radiomics score (see **Supplementary Figure S1**). The Random Forest (RF) algorithm was used to build the ultrasound radiomics model (Rad-model).

#### 2.5.3 Construction of the combined model

Based on the Multi-model and the Rad-model, a combined model (Com-model) was established by means of Logistic regression analysis and a nomogram was subsequently plotted.

### 2.6 Verification and clinical application of the nomogram

Receiver Operating Characteristic (ROC) curves were generated, and the AUC was calculated to assess the discriminatory performance of the model. Calibration curves were plotted, and the Hosmer-Lemeshow goodness-of-fit test was utilized to evaluate the calibration ability of the nomogram. The Brier score was computed to assess the overall performance of the model. Decision Curve Analysis (DCA) was constructed, and the clinical utility of the nomogram was estimated by calculating the net benefit across a range of threshold probabilities.

### 2.7 Rad-model assisted diagnosis

Fifty thyroid nodule images were randomly selected from enrolled patients and evaluated by 10 junior physicians (ultrasound diagnosis experience ≤3 years) and 10 senior physicians (ultrasound diagnosis experience >10 years) without knowledge of patient information. The 50 cases of thyroid nodules were assessed for benign and malignant conditions, as well as pathological results, and were re-evaluated one week later with the assistance of Rad-model prediction probability. The AUC was calculated to assess the diagnostic performance of both senior and junior physicians before and after the incorporation of ultrasound Rad-model assistance.

### 2.8 Statistical analyses

All statistical tests were conducted using SPSS 27.0, MedCalc (version 20.100), and R statistical software (version 4.0.2). We convert some continuous variables into categorical variables based on the ROC curve. Independent sample *t*-tests, chi-square tests, or Mann-Whitney *U* tests were employed to compare differences in age, nodule size, and multimodal ultrasound imaging characteristics between the training set and the test set. Logistic regression was utilized to construct Multi-model and Com-model, while the RF was adopted to establish Rad-model. ROC curves for the three models were generated using MedCalc. ROC analysis was performed, employing the Youden index to identify the optimal cut-off value for calculating the AUC, sensitivity, positive predictive value (PPV), negative predictive value (NPV) and accuracy. Internal validation of the models was performed using Bootstrap with 1000 resamplings. DeLong’s test was performed to compare the diagnostic performance of the models. A difference was considered statistically significant when *P* < 0.05. We utilized the “rms” and “pec” packages to build nomograms and calibration curves, the “caret” package for bootstrap validation, and the “ramda” and “ggDCA” packages to draw clinical decision curves for analysis using R statistical software.

## 3 Results

### 3.1 Characteristics of patients

This study included a total of 446 patients out of 490 patients with thyroid nodules. The set comprised 98 males and 348 females, with an average age of 50.09 ± 12.30 years. Based on the order of enrollment, the patients were divided into a training set (n=312) and a test set (n=134) in a ratio of 7:3. Within the training set, there were 172 benign cases and 140 malignant cases, while the test set contained 79 benign cases and 55 malignant cases. The maximum diameter of nodules in the training and test set was 10.10 ± 7.60 mm and 10.72 ± 7.30 mm. The baseline characteristics of the patients are presented in **Table 1**. There was no statistically significant difference between the two groups (P > 0.05).

**Table 1:** Baseline characteristics of patients in the training set and test set.

Characteristic	Training set (n = 312)	Test set (n = 134)	*P*
Age (years)	49.70 ± 14.04	50.79 ± 13.07	0.443
Gender			0.166
Male	63 (20.2%)	35 (26.1%)	
Female	249 (79.8%)	99 (73.9%)	
STE Mean (kPa)	32.82 ± 11.70	32.44 ± 11.94	0.445
STE Max (kPa)	63.69 ± 28.62	68.22 ± 38.03	0.476
STE Min (kPa)	14.93 ± 9.67	13.45 ± 8.78	0.353
STE SD (kPa)	9.39 ± 4.97	10.54 ± 6.59	0.391
SE Mean (%)	0.20 ± 0.11	0.19 ± 0.11	0.142
SE Max (%)	0.58 ± 0.70	0.53 ± 0.33	0.161
SE Min (%)	0.05 ± 0.06	0.06 ± 0.07	0.094
SE SD (%)	0.10 ± 0.08	0.10 ± 0.07	0.910
STQ Mean (kPa)	35.98 ± 16.12	34.62 ± 14.31	0.510
STQ Max (kPa)	61.68 ± 34.08	66.85 ± 43.08	0.524
STQ Min (kPa)	19.73 ± 13.39	17.76 ± 11.26	0.499
STQ SD (kPa)	9.43 ± 7.17	10.55 ± 8.97	0.656
Maximum diameter of nodules (mm)	10.10 ± 7.60	10.72 ± 7.30	0.063
Maximum diameter of nodules (<7.4 mm)	161 (51.6%)	57 (42.5%)	0.079
Multiplicity			0.422
solitary	272 (87.2%)	113 (84.3%)	
multiple	40 (12.8%)	21 (15.7%)	
Margin			0.970
smooth	132 (42.3%)	55 (41.0%)	
ill-defined	50 (16.0%)	22 (16.5%)	
irregular	130 (41.7%)	57 (42.5%)	
Halo sign			0.481
absent	288 (92.3%)	121 (90.3%)	
present	24 (7.7%)	13 (9.7%)	
Composition			0.557
cystic-solid	21 (6.7%)	9 (6.8%)	
solid	264 (84.6%)	109 (81.3%)	
predominantly solid	27 (8.7%)	16 (11.9%)	
Echogenicity			0.116
isoechoic	98 (31.4%)	48 (35.8%)	
markedly hypoechoic	45 (14.4%)	11 (8.2%)	
hypoechoic	164 (52.6%)	67 (50.0%)	
hyperechoic	1 (0.3%)	5 (3.7%)	
heterogeneous	4 (1.3%)	3 (2.3%)	
Echo texture			0.713
homogeneous	29 (9.3%)	11 (8.2%)	
heterogeneous	283 (90.7%)	123 (91.8%)	
Orientation			0.813
parallel	190 (60.9%)	80 (59.7%)	
vertical	122 (39.1%)	54 (40.3%)	
Calcification			0.774
absent	157 (50.3%)	73 (54.5%)	
coarse calcification	25 (8.0%)	13 (9.7%)	
microcalcification	101 (32.4%)	38 (28.3%)	
indeterminate punctate echogenic foci	28 (9.0%)	10 (7.5%)	
peripheral calcification	1 (0.3%)	0 (0%)	
Relationship to capsule			0.540
>2 mm	118 (37.8%)	48 (36.1%)	
≤2 mm	163 (52.2%)	68 (50.4%)	
extracapsular extension	31 (10.0%)	18 (13.5%)	
CDFI			0.398
0	135 (43.3%)	51 (38.1%)	
I	123 (39.4%)	63 (47.0%)	
II	44 (14.1%)	18 (13.4%)	
III	10 (3.2%)	2 (1.5%)	
Benign and malignant			0.455
benign	172 (55.1%)	79 (59.0%)	
malignant	140 (44.9%)	55 (41.0%)	

Data are expressed as number of patients (%) unless otherwise specified. Percentages may not sum to 100% due to rounding. All discrepancies were cross-verified with raw data and corrected.

### 3.2 Construction and performance of the model

#### 3.2.1 The Multi-model

Univariate logistic regression analyses showed that age (≤51 years) (OR: 2.863, 95% CI: 1.796-4.563), gender (female) (OR: 0.496, 95% CI: 0.283-0.869), STE Mean (OR: 1.046, 95% CI: 1.024-1.069), STE Max (OR: 1.012, 95% CI: 1.004-1.021), STE Min (OR: 1.025, 95% CI: 1.001-1.050), STE SD (OR: 1.064, 95% CI: 1.015-1.115), SE Mean (OR: 0.058, 95% CI: 0.006-0.596), STQ Mean (OR: 1.026, 95% CI: 1.011-1.041), STQ Max (OR: 1.011, 95% CI: 1.003-1.019), STQ SD (OR: 1.064, 95% CI: 1.026-1.103), maximum diameter (≤7.4 mm) (OR: 1.851, 95% CI: 1.177-2.903), margin (ill-defined) (OR: 9.704, 95% CI: 4.584-20.593), margin (irregular) (OR: 13.382, 95% CI: 7.254-24.684), halo sign (present) (OR: 0.224, 95% CI: 0.075-0.670), composition (solid) (OR: 9.792, 95% CI: 2.236-42.882), echogenicity (markedly hypoechoic) (OR: 3.219, 95% CI: 1.861-5.569), echogenicity (hypoechoic) (OR: 4.809, 95% CI: 2.261-10.229), orientation (vertical) (OR: 7.875, 95% CI: 4.697-13.203), calcification (coarse calcification) (OR: 0.218, 95% CI: 0.085-0.558), calcification (microcalcification) (OR: 0.204, 95% CI: 0.119-0.350) is a risk factor for PTC.

**Figure 3:**
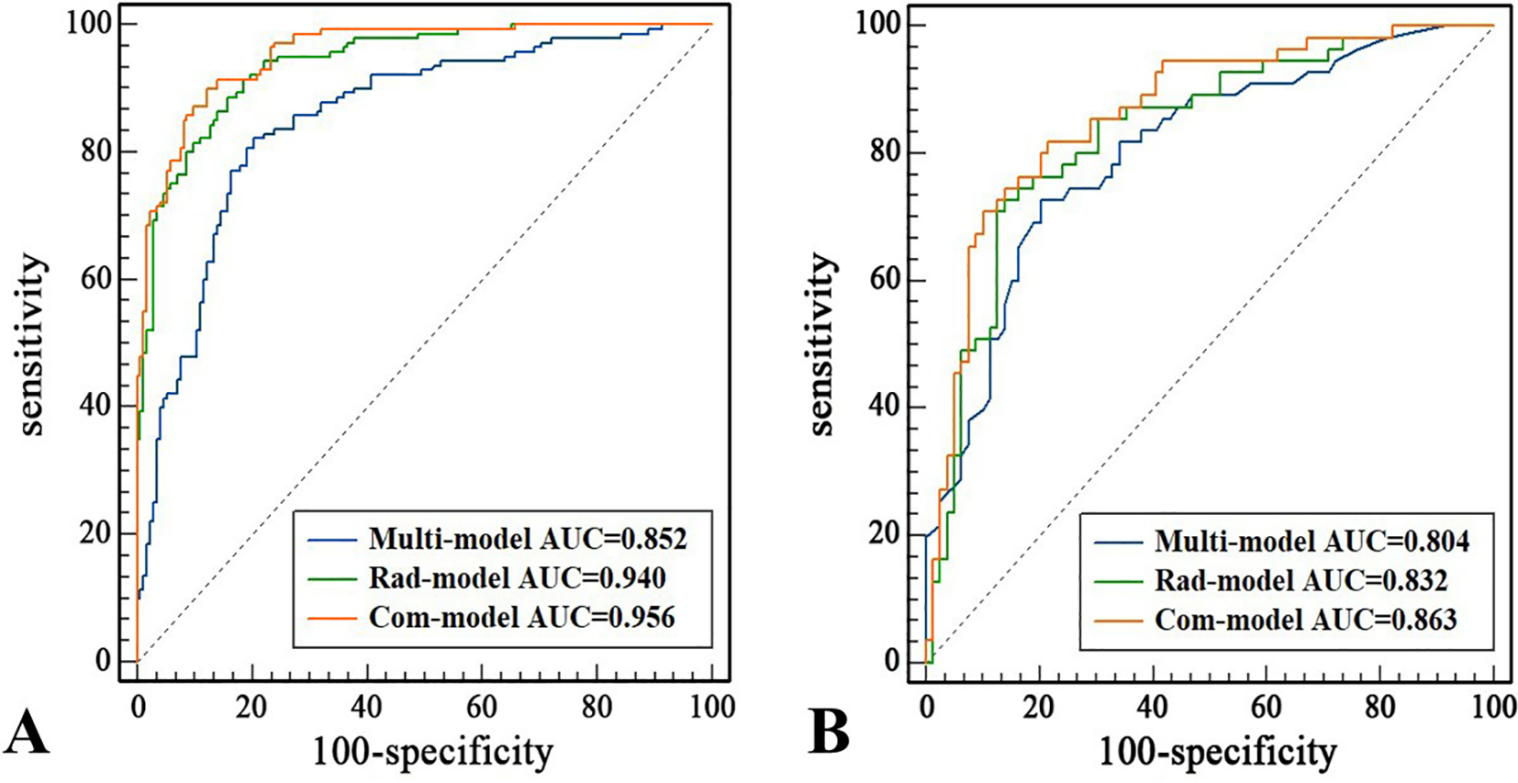
ROC Curves of the Com-Model in the Training **(A)** and Test Set **(B)**.

Multivariate logistic regression analysis showed that age (≤51 years) (OR: 2.752, 95% CI: 1.546-4.900), STE Mean (OR: 1.036, 95% CI: 1.010-1.062), margin (ill-defined) (OR: 6.187, 95% CI: 2.700-14.178), and margin (irregular) (OR: 7.011, 95% CI: 3.545-13.865), orientation (vertical) (OR: 3.515, 95% CI: 1.926-6.415) were independent risk factors for PTC (**Table 2**). The AUC of the Multi-model in the training set was 0.852 (95% CI: 0.808-0.890), sensitivity was 82.14%, specificity was 79.65%, PPV was 76.67%, and NPV was 84.57%. In the test set, the AUC of the Multi-model was 0.804 (95% CI: 0.726-0.867), sensitivity was 72.73%, specificity was 79.75%, PPV was 71.43%, and NPV was 80.77% (**Figure 3** and **Table 3**).

**Table 2:** Results based on univariate and multivariate logistic regression analysis of the training set.

Characteristic	Univariate analysis		Multivariate analysis	
	95% CI	*P*	95% CI	*P*
Age ≤ 51 (years)	1.796-4.563	<0.001	1.546-4.900	<0.01
Gender				
Male	Reference			
Female	0.283-0.869	0.014		
STE Mean (kPa)	1.024-1.069	<0.001	1.010-1.062	0.006
STE Max (kPa)	1.004-1.021	0.004		
STE Min (kPa)	1.001-1.050	0.037		
STE SD (kPa)	1.015-1.115	0.009		
SE Mean (%)	0.006-0.596	0.017		
SE Max (%)	0.386-1.246	0.221		
SE Min (%)	0.001-1.044	0.053		
SE SD (%)	0.006-2.622	0.181		
STQ Mean (kPa)	1.011-1.041	<0.001		
STQ Max (kPa)	1.003-1.019	0.004		
STQ Min (kPa)	0.998-1.032	0.091		
STQ SD (kPa)	1.026-1.103	<0.001		
Maximum diameter of nodules (mm)	0.940-1.001	0.057		
Maximum diameter of nodules (<7.4 mm)	1.177-2.903	0.008		
Multiplicity				
solitary	Reference			
multiple	0.405-1.564	0.508		
Margin				
smooth	Reference			
ill-defined	4.584-20.539	<0.001	2.700-14.178	<0.01
irregular	7.254-24.684	<0.001	3.545-13.865	<0.01
halo sign				
absent	Reference			
present	0.075-0.670	0.007		
Composition				
cystic-solid	Reference			
solid	2.236-42.882	0.002		
predominantly solid	0.272-10.024	0.585		
Echogenicity				
isoechoic	Reference			
markedly hypoechoic	1.861-5.569	<0.001		
hypoechoic	2.261-10.229	<0.001		
hyperechoic	0	1.000		
heterogeneous	0.097-9.789	0.982		
Echo texture				
homogeneous	Reference			
heterogeneous	0.843-4.351	0.121		
Orientation				
parallel	Reference			
vertical	4.697-13.203	<0.001	1.926-6.415	<0.01
Calcification				
absent	Reference			
coarse calcification	0.085-0.558	0.001		
microcalcification	0.119-0.350	<0.001		
indeterminate punctate echogenic foci	0.228-1.256	0.151		
peripheral calcification	0	1.000		
Relationship to capsule				
>2 mm	Reference			
≤2 mm	0.522-1.355	0.476		
extracapsular extension	0.553-2.697	0.620		
CDFI				
0	Reference			
I	0.607-1.615	0.968		
II	0.282-1.166	0.125		
III	0.118-1.917	0.296		

**Table 3:** Predictive performance of the three models in the training set and test set.

Variables	The Multi-model		The Rad-model		The Com-model	
	Training set	Test set	Training set	Test set	Training set	Test set
AUC	0.852	0.804	0.940	0.832	0.956	0.863
95% CI	0.808-0.890	0.726-0.867	0.907-0.963	0.758-0.891	0.926-0.976	0.793-0.917
Sensitivity (%)	82.14	72.73	88.57	72.73	90.00	70.91
Specificity (%)	79.65	79.75	84.30	86.08	97.79	89.87
PPV (%)	76.67	71.43	82.12	78.43	96.92	82.98
NPV (%)	84.57	80.77	90.06	81.93	92.31	81.61
FNR (%)	17.86	27.27	11.43	27.27	10.00	29.09

#### 3.2.2 The Rad-model

**Figure 4:**
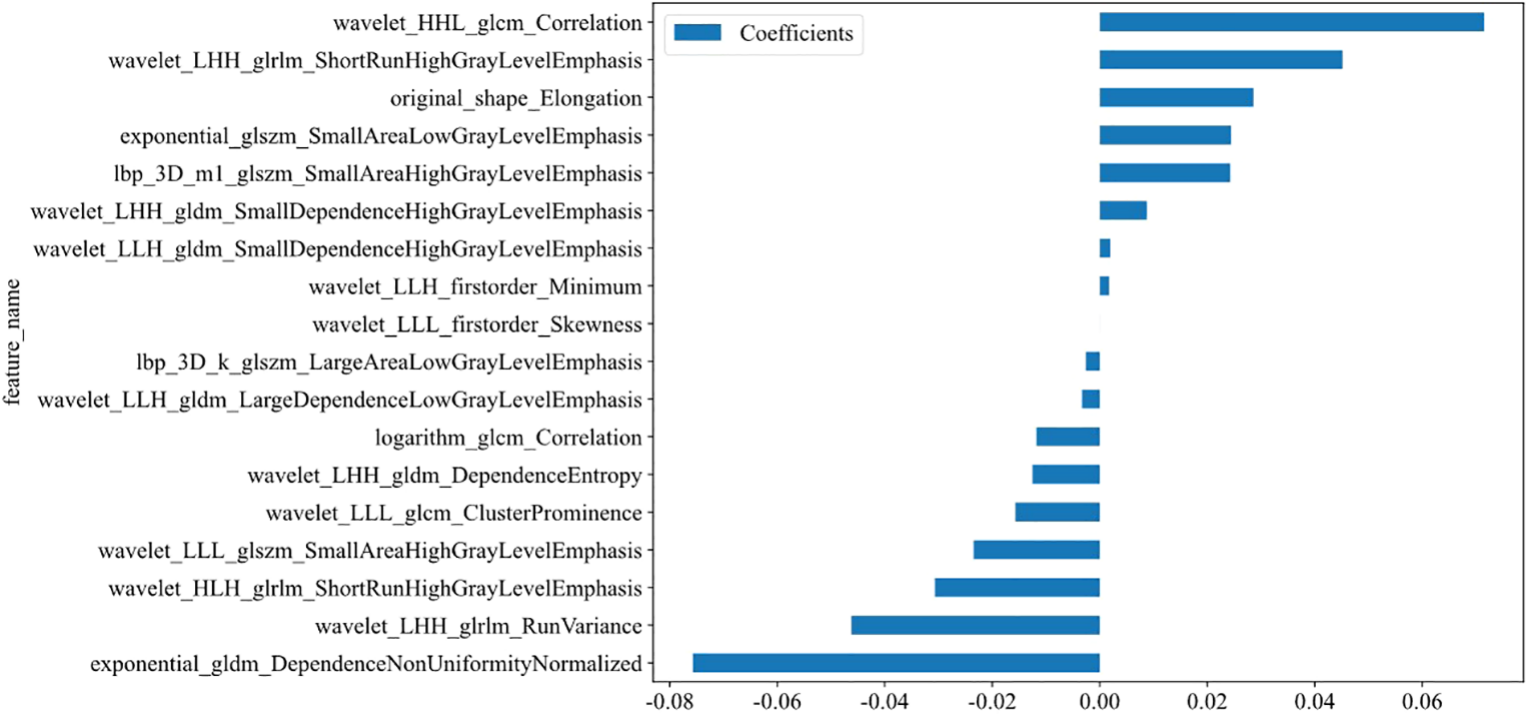
Coefficients of the 17 selected features (see **Supplementary Figure S2**).

A total of 1,562 features related to radiomics were extracted initially. After applying dimensionality reduction, 17 features were selected (**Figure 4**), and the Rad-model was developed using the RF algorithm. The AUC of the Rad-model in the training set was 0.940 (95% CI: 0.907-0.963), sensitivity was 88.57%, specificity was 84.30%, PPV was 82.12%, and NPV was 90.06%. In the test set, the AUC of the Rad-model was 0.832 (95% CI: 0.758-0.891), sensitivity was 72.73%, specificity was 86.08%, PPV was 78.43%, and NPV was 81.93% (**Figure 3** and **Table 3**).

#### 3.2.3 The Com-model

The Com-model was developed by integrating the Multi-model and the Rad-model. The AUC of the Com-model in the training set was 0.956 (95% CI: 0.926-0.976), sensitivity was 90.00%, specificity was 97.79%, PPV was 96.92%, and NPV was 92.31%. Internal validation was performed using 1000 bootstrap resampling iterations, resulting in an average AUC of 0.954 for the Com-model. In the test set, the AUC of the Com-model was 0.863 (95% CI: 0.793-0.917), sensitivity was 70.91%, specificity was 89.87%, PPV was 82.98%, and NPV was 81.61% (**Figure 3** and **Table 3**).

### 3.3 Verification and clinical application of the nomogram

**Figure 5:**
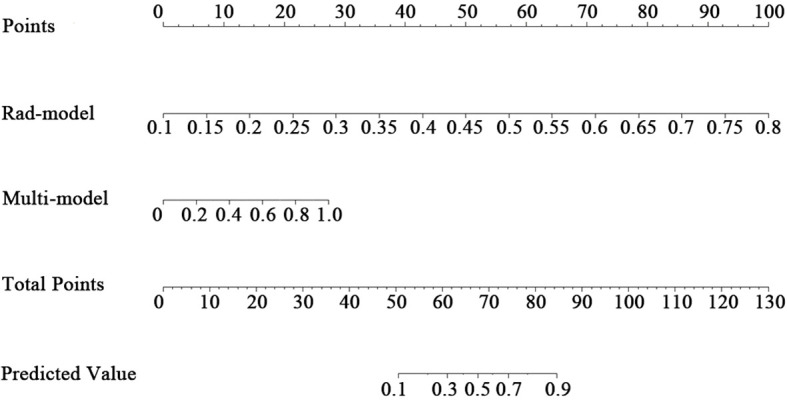
Nomogram of the Com-Model.

**Figure 6:**
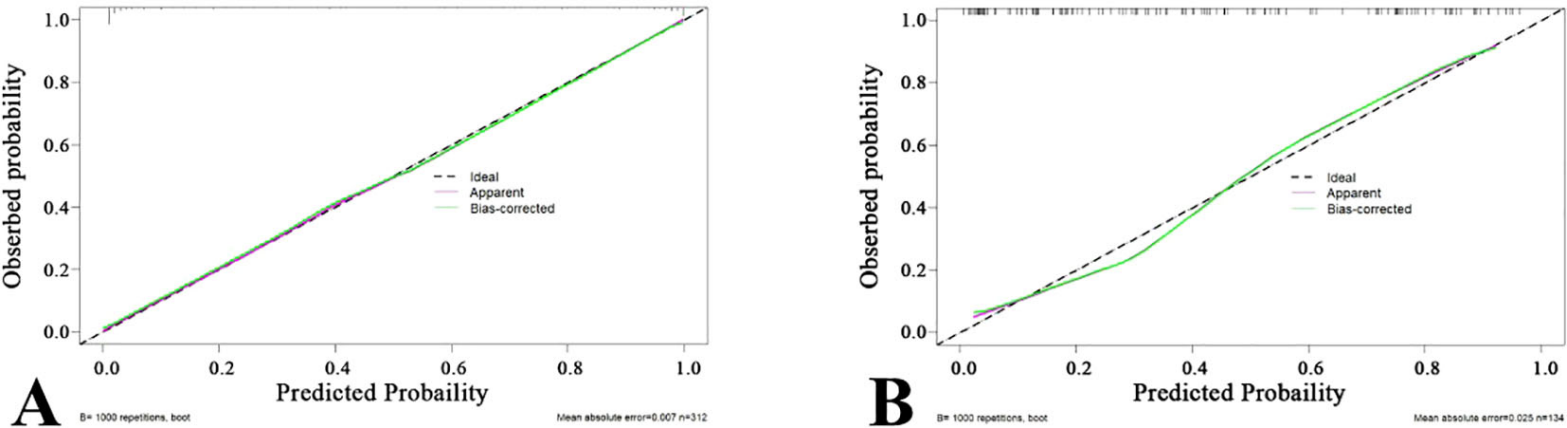
Calibration Curves of the Com-Model in the Training Set **(A)** and Test Set **(B)**.

**Figure 7:**
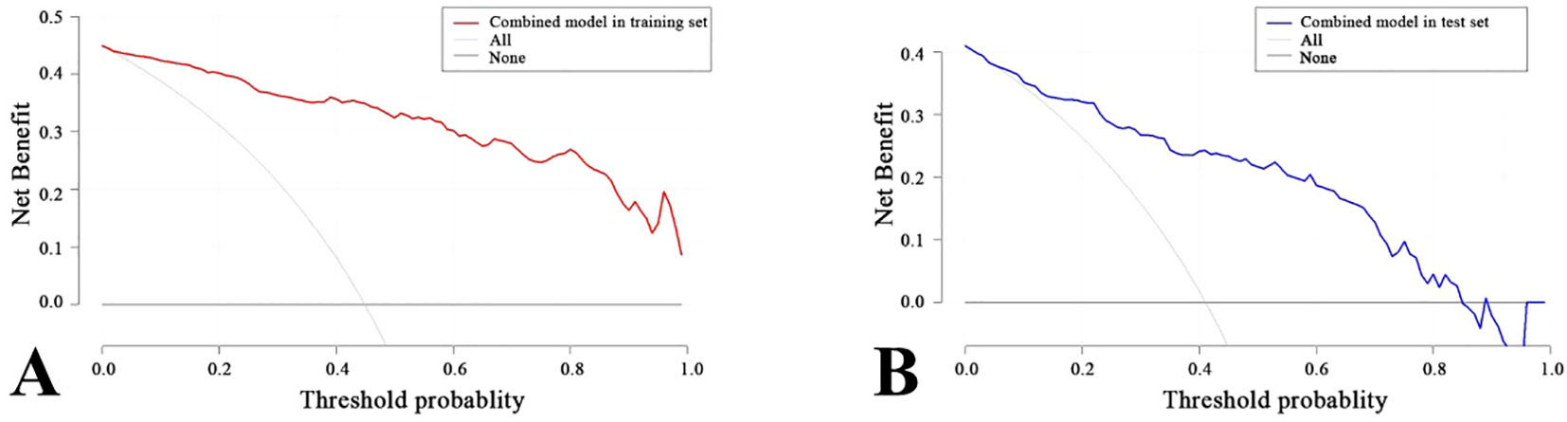
Clinical Decision Curves of the Com-Model in the Training Set **(A)** and Test Set **(B)**.

A nomogram was created to estimate the probability of PTC. Using the nomogram derived total score, we stratified patients into low (≤54, malignancy probability ≤15%), intermediate (54-68, malignancy probability15-50%), and high-risk (>68, malignancy probability >50%) categories. For high-risk nodules, immediate FNA or surgery is recommended. Intermediate-risk cases may benefit from selective FNA guided by clinical factors, while low-risk nodules warrant surveillance, avoiding unnecessary biopsies. (**Figure 5**). The calibration plot in **Figure 6** illustrates the comparison between the predicted positive rate derived from the nomogram and the actual observations. According to the Hosmer-Lemeshow goodness-of-fit test, both the training set (*P* = 0.913) and the test set (*P* = 0.854) show a strong fit. The Brier scores for the training and test sets are 0.08 and 0.15, respectively, indicating that our prediction model demonstrates overall robustness. The DCA shows that the model offers considerable advantages within the range of 0.15 to 0.90 (**Figure 7**). To further assess the effectiveness of the models, we performed statistical comparisons of the ROC curves using DeLong’s test (**Table 4**). The findings indicate that in the training set, the Com-model was the most effective. In the test set, the Com-model surpassed the Multi-model, with no significant difference noted when compared to the ultrasound Rad-model.

**Table 4:** Delong test of three models.

Variables	The Multi-model - The Rad-model		The Rad-model - The Com-model		The Com-model - The Multi-model	
	Training set	Test set	Training set	Test set	Training set	Test set
*Z*	3.64	0.65	2.42	1.55	5.34	2.16
*P*	<0.001	0.52	0.02	0.12	<0.001	0.03

### 3.4 Effectiveness of the Rad-model assistance

**Figure 8:**
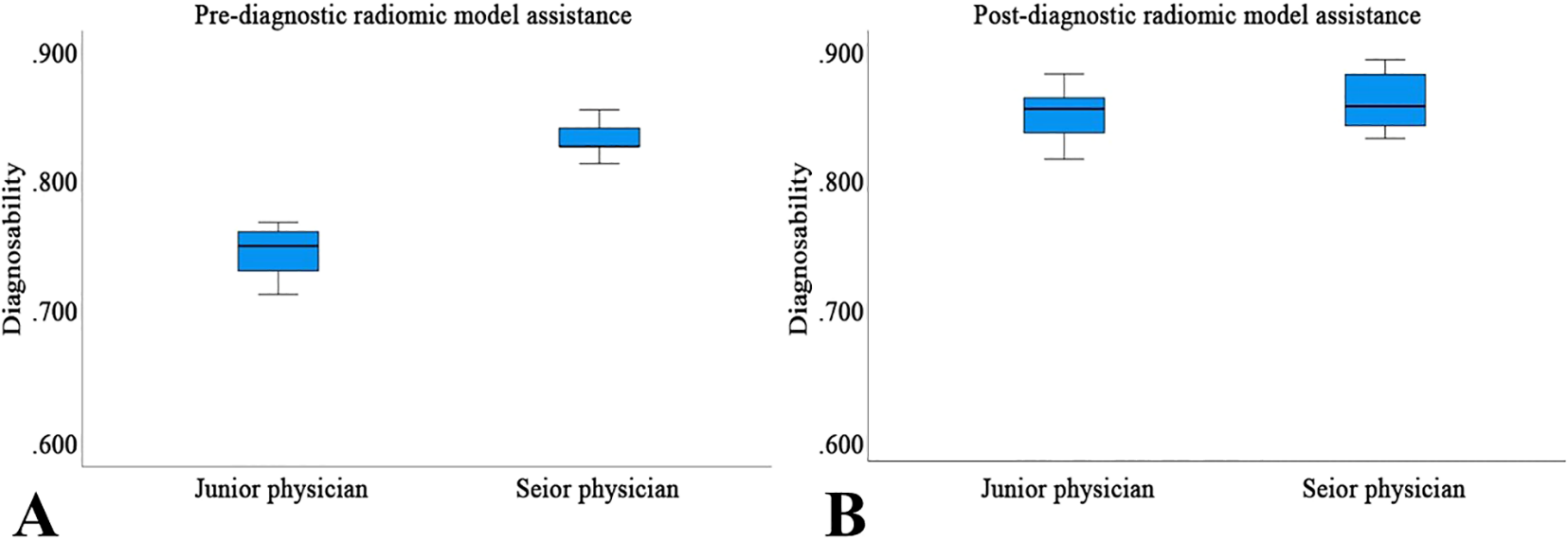
The performance of Pre- **(A)** and Post-diagnostic **(B)** Radiomic Model Assistance of junior and senior physicians.

Before utilizing the Rad-model for diagnostic assistance, the AUC for junior physicians was 0.748, while that for senior physicians was 0.837 (**Table 5** and **Figure 8**). Following the implementation of the Rad-model, the average AUC for junior physicians significantly increased to 0.851, and for senior physicians, it improved to 0.862. DeLong’s test indicated a significant enhancement in the diagnostic performance of junior physicians after model assistance (<0.001), suggesting that the Rad-model effectively shortened their learning curve. Furthermore, there was no significant difference in AUC between senior and junior physicians after receiving model assistance, indicating that the model successfully narrowed the diagnostic gap between physicians with varying levels of experience (**Table 6**).

**Table 5:** The performance of Pre- and Post-diagnostic Radiomic Model Assistance of junior and senior physicians.

Variables	Pre-diagnostic radiomic model assistance	Post-diagnostic radiomic model assistance
junior physicians (AUC)	0.748	0.851
Sensitivity (%)	80.12	85.63
Specificity (%)	82.71	87.24
PPV (%)	78.97	86.61
NPV (%)	83.82	86.98
senior physicians (AUC)	0.837	0.862
Sensitivity (%)	68.23	85.54
Specificity (%)	76.31	85.47
PPV (%)	71.43	83.02
NPV (%)	73.58	84.92

**Table 6:** The DeLong test for comparing Pre- and Post-diagnostic Radiomic Model Assistance of junior and senior physicians.

Variables	Pre- and Post-diagnostic		Post-diagnostic
	junior physicians	senior physicians	junior-senior physicians
*Z*	3.12	1.72	0.65
*P*	<0.001	0.08	0.51

## 4 Discussion

This study integrated multimodal ultrasound imaging features with ultrasound radiomics analysis to develop and validate a Com-model for the preoperative differentiation of benign and malignant thyroid nodules classified as C-TIRADS 4A. Experimental data demonstrated that the model achieved an AUC of 0.956 in the training set and 0.863 in the test set, showcasing reliable diagnostic performance. This model enables rapid and accurate differentiation of benign and malignant C-TIRADS 4A thyroid nodules, thereby effectively reducing the risk of unnecessary biopsies for benign nodules.

This study confirmed that age is an independent risk factor for PTC. The results showed that the risk of malignancy in nodules was significantly higher in patients aged ≤51 years, a finding consistent with the research by Wang et al. (14) This phenomenon may be attributed to increased public health awareness and the widespread adoption of regular health checkups, enabling more cases to be identified at an early stage of the disease. Additionally, advancements in modern diagnostic techniques have significantly improved the detection rate of PTC. The study included only patients aged 18 and above, as the number of patients under 18 undergoing thyroid surgery in multicenter institutions was minimal (most of these patients had already received treatment in pediatric specialty hospitals). The exclusion of adolescent patients aimed to avoid analytical bias caused by potential biological differences between adults and adolescents, thereby ensuring the accuracy of the research data and the validity of the conclusions.

A higher ultrasound elasticity score indicates a greater likelihood of malignancy in nodules (15, 16), reflecting that malignant nodules typically exhibit higher stiffness compared to benign ones. Research by Luo et al. (17) demonstrated that the combination of SWE with American College of Radiology Thyroid Imaging Reporting and Data System (ACR TI-RADS) significantly improves the efficiency and accuracy of diagnosing thyroid nodule characteristics. Similarly, Zhang et al. (18) found that adding elastography to multimodal ultrasound for predicting the benign or malignant nature of C-TIRADS 4A nodules significantly enhances diagnostic consistency, sensitivity, and specificity, outperforming any single method. In this study, STE Mean is an important risk factor. This result further validates the effectiveness and importance of ultrasound elastography in assisting the differentiation of benign and malignant thyroid nodules. Specifically, by integrating ultrasound elastography with various other ultrasound features, the model developed in this study not only improves the accuracy of thyroid nodule characterization but also enhances the reliability of clinical decision-making, helping to reduce unnecessary invasive procedures.

Vertical orientation (where the longitudinal growth of the nodule exceeds its transverse growth) is considered an independent risk factor for malignant thyroid nodules (19, 20). From a histological perspective, this phenomenon may be attributed to the active division of tumor cells in the anterior-posterior direction within malignant nodules, while remaining relatively quiescent in other directions. This aggressive growth pattern aims to increase the tumor’s surface area, thereby facilitating more efficient nutrient acquisition and accelerating its growth process.

Additionally, blurred or irregular margins are one of the critical imaging features for assessing the nature of thyroid nodules (21). Studies have shown that the BRAF V600E mutation is the most prevalent genetic alteration in PTC, present in approximately 60–80% of cases. This mutation constitutively activates the MAPK/ERK signaling pathway, promoting uncontrolled cell proliferation and tumorigenesis and promotes the invasiveness and metastatic potential of tumor cells, thereby influencing the morphological appearance of the nodules. Specifically, it leads to blurred, jagged, or spiculated margins, which are often indicative of malignancy. The BRAF V600E mutation has been confirmed to be associated with the high aggressiveness and poor prognosis of PTC (21, 22).

The Com-model achieved an AUC of 0.863 in the test set, outperforming standalone C-TIRADS 4A classification [reported AUC: 0.70-0.82 in prior studies (5, 6)] and ACR TI-RADS [AUC: 0.76-0.85 (5, 18)]. For instance, Zhang et al. (18) reported an AUC of 0.834 for ACR TI-RADS in differentiating C-TIRADS 4A nodules, while our Com-model achieved higher specificity (89.87% vs. 76.5%) and comparable sensitivity (70.91% vs. 72.1%). Notably, the integration of radiomics and multimodal ultrasound features enabled our model to capture subtle malignant characteristics (e.g., microstructural heterogeneity) that conventional systems may overlook. Compared to AI-driven approaches, such as TNet [AUC: 0.865 (23)] and the CNN-based framework by Tao et al. [AUC: 0.872 (16)], our Com-model demonstrated superior generalizability across multicenter data. However, unlike deep learning models requiring large annotated datasets, our radiomics framework relies on interpretable handcrafted features, aligning better with clinical workflows.

This study compared the diagnostic performance of senior and junior physicians in differentiating thyroid nodules before and after the application of the Rad-model. Although junior physicians lack extensive experience in ultrasound diagnosis, their diagnostic accuracy significantly improved with the support of the Rad-model, reaching a level comparable to that of senior physicians. This not only greatly shortened the learning curve for junior physicians, accelerating their path to professional proficiency, but also enhanced the overall efficiency and accuracy of thyroid nodule diagnosis. These findings suggest that the Rad-model can effectively address the challenges posed by limited clinical experience, providing robust technical support for younger physicians and thereby improving the overall quality of healthcare services.

The Com-model exhibited a false-negative rate of 10.00% in the training set and 29.09% in the test set, indicating a non-negligible risk of missing malignant cases, particularly in test set. While this rate aligns with prior studies [e.g., Zhang et al. (24)]. To mitigate the risks of false-negative results, we advocate for a tiered follow-up protocol combining short-interval ultrasound surveillance and clinical risk stratification: 6 months follow-up for intermediate-risk cases and 12 months reassessment for low-risk cases. Future iterations of the model will integrate dynamic imaging biomarkers and molecular testing to further reduce FNR, ensuring early intervention for initially missed malignancies.

Future research will focus on integrating molecular and biochemical profiling (25) with our imaging-based model to achieve a holistic assessment of thyroid nodules. For instance, combining radiomic heterogeneity with BRAF V600E or TERT promoter mutations could improve the identification of aggressive PTC subtypes, while metabolic markers of oxidative stress may refine angioinvasion risk prediction (26). Such multi-omics fusion aligns with the goals of precision oncology, enabling tailored surveillance and treatment strategies for borderline or ambiguous nodules. Additionally, liquid biopsy-derived biomarkers (e.g., ctDNA) (27) could complement ultrasound surveillance by providing real-time molecular insights into nodule dynamics. This approach may bridge the gap between static imaging assessments and the evolving biological behavior of thyroid malignancies.

To ensure consistency across multiple centers, all participating centers use the same ultrasound system and follow standardized imaging protocols. In the future, we will collaborate with medical institutions using different ultrasound systems (such as GE Logiq, Philips EPIQ, Siemens Acuson) to collect external datasets to ensure their applicability in the real world.

This study has several notable limitations. First, while it was impossible to completely eliminate all subjective factors during the analysis of multimodal ultrasound features, we addressed this by involving multiple evaluators and applying consistency tests to correct for potential subjective biases. Second, some cases relied on cytopathological reports, which inherently carry a certain rate of false negatives. To mitigate this issue, we incorporated BRAF gene testing results and conducted a 6 months follow-up observation of patients to more accurately assess the nature of the nodules.

## 5 Conclusion

In summary, we successfully developed a Com-model that integrates the Multi-model with the Rad-model to differentiate between benign and malignant thyroid nodules. The results demonstrate that this Com-model exhibits significant advantages in distinguishing C-TIRADS 4A thyroid nodules, providing physicians with a rapid and accurate risk assessment tool. It effectively identifies potential malignant lesions while reducing unnecessary invasive examinations or treatments for patients with benign nodules.

## Data Availability

The original contributions presented in the study are included in the article/**Supplementary Material**. Further inquiries can be directed to the corresponding author.
