# Supplementary material for: The value of a combined model based on ultra-radiomics and multi-modal ultrasound in the benign-malignant differentiation of C-TIRADS 4A thyroid nodules: a prospective multicenter study
Source: Front Oncol. 2025 May 8;15:1543020. doi: 10.3389/fonc.2025.1543020 (PMC12095005; doi:10.3389/fonc.2025.1543020)

ELECTRONIC SUPPLEMENTARY MATERIAL

The Value of a Combined Model Based on Ultra-Radiomics and Multi-modal Ultrasound in the Benign-Malignant Differentiation of C-TIRADS 4A Thyroid Nodules: A Prospective Multicenter Study

**Supplementary Fig. S1** The formula of radiomics score


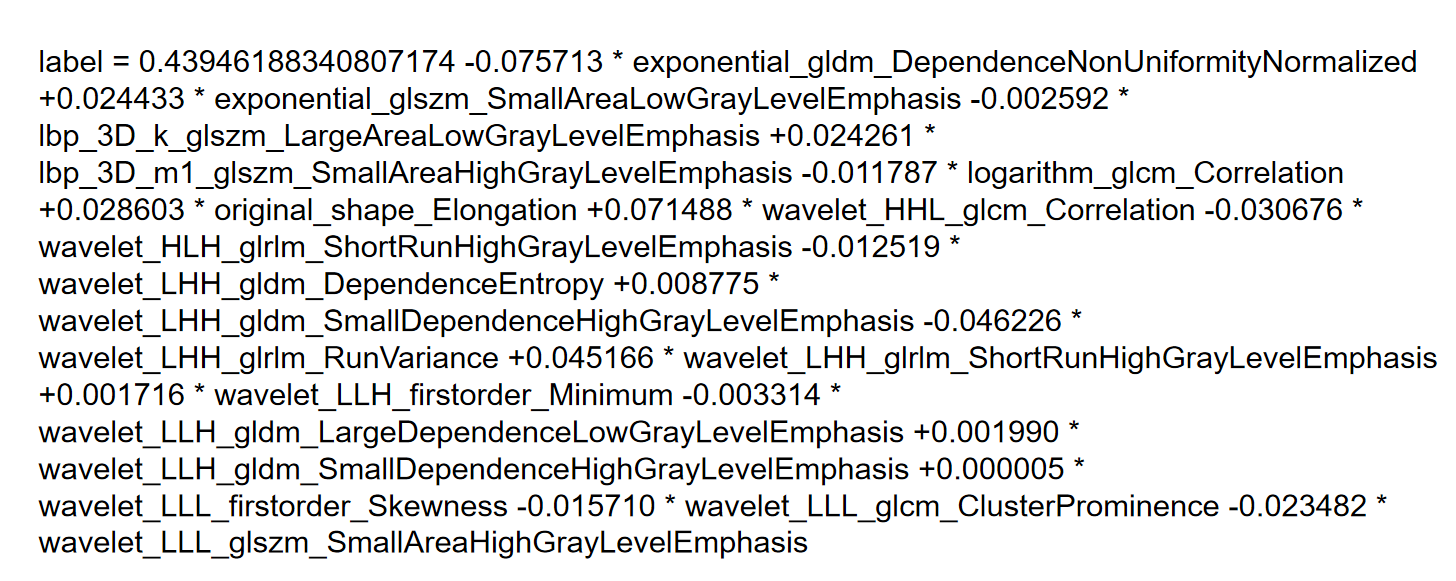


**Supplementary Fig. S2** Coefficients of the 17 selected features


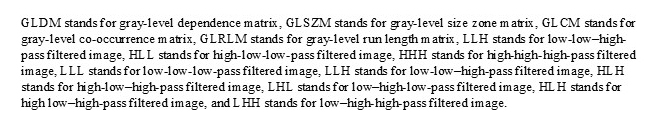

Supplement: Supplementary file 1 [file DataSheet1.docx]
